# Utilizing the folate receptor for active targeting of cancer nanotherapeutics

**DOI:** 10.3402/nano.v3i0.18496

**Published:** 2012-12-07

**Authors:** Grant L. Zwicke, G. Ali Mansoori, Constance J. Jeffery

**Affiliations:** 1 Department of Biological Sciences, University of Illinois at Chicago, Chicago, IL, USA; 2 Department of Bioengineering, University of Illinois at Chicago, Chicago, IL, USA; 3 Department of Chemical Engineering, University of Illinois at Chicago, Chicago, IL, USA; 4 Department of Physics, University of Illinois at Chicago, Chicago, IL, USA

**Keywords:** cancer, conjugates, doxorubicin, folate, folic acid, folate receptor, gold, nanoaggregates, ^99m^Tc-EC20, nanotechnology

## Abstract

The development of specialized nanoparticles for use in the detection and treatment of cancer is increasing. Methods are being proposed and tested that could target treatments more directly to cancer cells, which could lead to higher efficacy and reduced toxicity, possibly even eliminating the adverse effects of damage to the immune system and the loss of quick replicating cells. In this mini-review we focus on recent studies that employ folate nanoconjugates to target the folate receptor. Folate receptors are highly overexpressed on the surface of many tumor types. This expression can be exploited to target imaging molecules and therapeutic compounds directly to cancerous tissues.

The main problem with cancer treatments today is that they involve a balancing act between the destruction of cancerous tissue and the destruction of healthy tissues, including damage to the immune system and highly replicating cells (gastrointestinal epithelia and hair follicles). As of now, a patient's prognosis hinges on the discovery of cancerous cells early enough and in a stage that is manageable for possible treatment. Newly developed nanotechnological techniques are bringing hope to the world of oncologic research. Currently, nanotechniques are being tested and used for the improvement of current technologies and for the development of new ones, in cancer detection, prevention, and treatment.

It is the innate qualities of nanoparticles that make them advantageous for use in cancer management. Nanoscale particles have a maximum surface to volume ratio making them perfect for surface functionalization and conjugation with therapeutic agents. Also, due to their size and malleable surface properties, nanoparticles can be synthesized to use passive or active targeting systems with superior tumor specificity than current drug methods (1, 2).

Passive targeting takes advantage of differences between cancer cells and healthy cells and tissues. Two of the differences include a leaky vasculature and an acidic tumor microenvironment (2). Cancer cells grow rapidly, which requires speedy vascularization leading to a defective vascular architecture that is more permeable to macromolecules than normal tissues. This increased permeability makes cancerous tissues easily accessible to chemotherapeutic drugs (Fig. 1), and with the lack of lymphatic drainage in the tumor bed, the result is an accumulation of the drug within cancer cells (2). This effect, called the enhanced permeability and retention (EPR) effect, also affects the ability of a cell to uptake vital nutrients and oxygen (3). It is a response to overcome limited cell respiration that results in nanoparticles having the ability to penetrate interstitial tumor vasculature with higher retention times than regular tissues (4, 5). The EPR effect is important as it is observed in all nanoparticle categories (6).

**Fig. 1:**
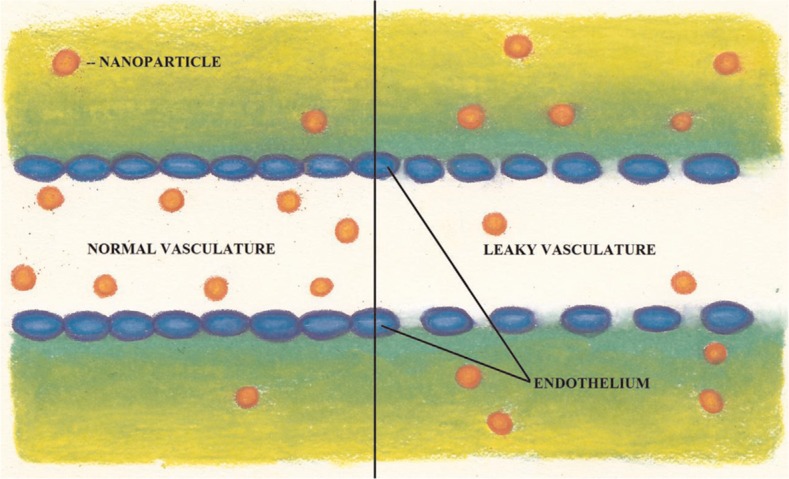
The vasculature of cancerous tissues can be more leaky than that of healthy tissues. This difference in leakiness can be exploited in anticancer drug delivery. Vasculature of the tissue on the left is normal while the tissue on the right is leaky. The nanoparticles passively enter through the leaky vasculature into the tumor tissue due to the enhanced permeability and retention (EPR) effect. (Illustrated by Samantha L. Lindberg).

The microenvironment of a tumor is another aid to passive targeting. Within cancer cells, the cytosol tends to be acidic (7). This low pH can be exploited by the use of pH sensitive drug conjugates that are degraded after entering into the cancer cells, resulting in a release of the active drug into target tissues (7). In direct local application, a drug is applied directly to tumor tissue, avoiding systemic circulation (2). This permits an increased concentration of the drug to be applied in cancer cells without a high general toxicity (7).

Nanoparticles can also be used in the active targeting of cancer cells. In active targeting, a nanoparticle is typically conjugated with a targeting moiety, thereby permitting preferential accumulation of the drug within selected tissues, individual cancer cells, or intracellular organelles that are associated with specific molecules in cancer cells (2). Molecular targets for active targeting methods in proposed cancer treatments include cell-surface carbohydrates (carbohydrate targeting), cellular antigens for antibodies (antibody targeting), and cell surface receptors (receptor targeting) (2). In carbohydrate-directed targeting, nanotreatments are targeted to tumor cells by attaching them to lectins or other proteins that bind to cell-surface carbohydrates (2). Antibody targeting similarly makes use of the frequently observed overexpression of many antigens on tumor cell surfaces. Receptor targeting involves attaching drugs to a ligand that binds to receptors expressed on the cell surface, thereby initiating receptor-mediated endocytosis. In some studies the high-affinity receptor for the vitamin folic acid (the folate receptor) has been used as a target (8). The folate receptor appears to be a promising target for cancer treatment and detection and will be discussed in more detail below.

A variety of nanoparticle materials are being studied for use in drug targeting. Nanoparticles consisting of polymer–drug conjugates display an EPR effect (2). Polymer–drug conjugates include polymeric nanoparticles, micelles, and dendrimers (Fig. 2). In polymeric nanoparticles, drugs are conjugated to the side chain of a linear polymer through a linker that contains a cleavable bond (7). Inside the cancerous tissue, the linker is cleaved and the drug becomes active. Polymeric micelles have a nano-sized core/shell structure. The hydrophobic core region serves as a reservoir for drugs. The outer hydrophilic shell stabilizes the core and renders the overall complex to be water-soluble, which makes the micelle appropriate for intravenous administration. A dendrimer is a synthetic polymeric macromolecule of nanometer dimensions that has multiple highly branched chains emerging radially from a central core and has many sites of potential drug attachment (7).

**Fig. 2:**
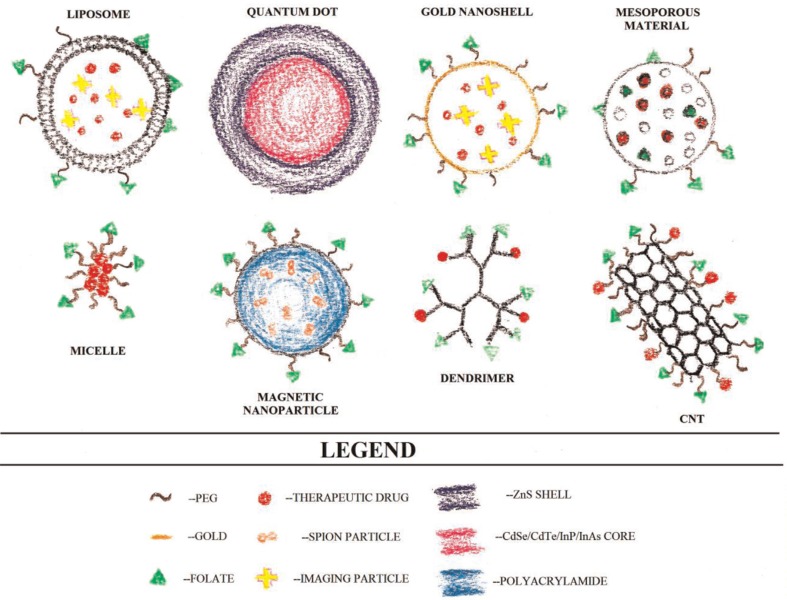
A variety of nanoparticle materials are being studied for use in imaging and drug targeting. These include liposomes, quantum dots (QD), gold-nanoshells, mesoporous materials, micelles, magnetic nanoparticles, dendrimers, and CNTs. (Illustrated by Grant Zwicke).

Other proposed cancer drug delivery methods make use of liposomes, viral nanoparticles, or carbon nanotubes (CNTs) (Fig. 2). Liposomes are spherical structures composed of an outer lipid bilayer surrounding a central aqueous space (7). Viral nanoparticles are derived from a variety of viruses, including the cowpea mosaic virus, cowpea chlorotic mottle virus, canine parvovirus, and bacteriophages (7). An advantage of viral nanoparticles is that recombinant deoxyribonucleic acid (DNA) methods can be used to cause the surface display of the targeting molecules (7). CNTs, despite that fact that they need modification to make them water-soluble, have the advantage of being able to be conjugated to a wide variety of active molecules such as nucleic acids, peptides, proteins, and other therapeutic compounds (7). CNTs can even be functionalized with multiple molecules at one time, which make them advantageous for cancer treatment. Fluorescently linked CNTs can also be used to detect cancerous cells. In one *in vivo* study, drugs bound to CNTs were shown to be more effectively internalized into cells than the free drug (7).

## Folate and the folate receptor

**Fig. 3:**
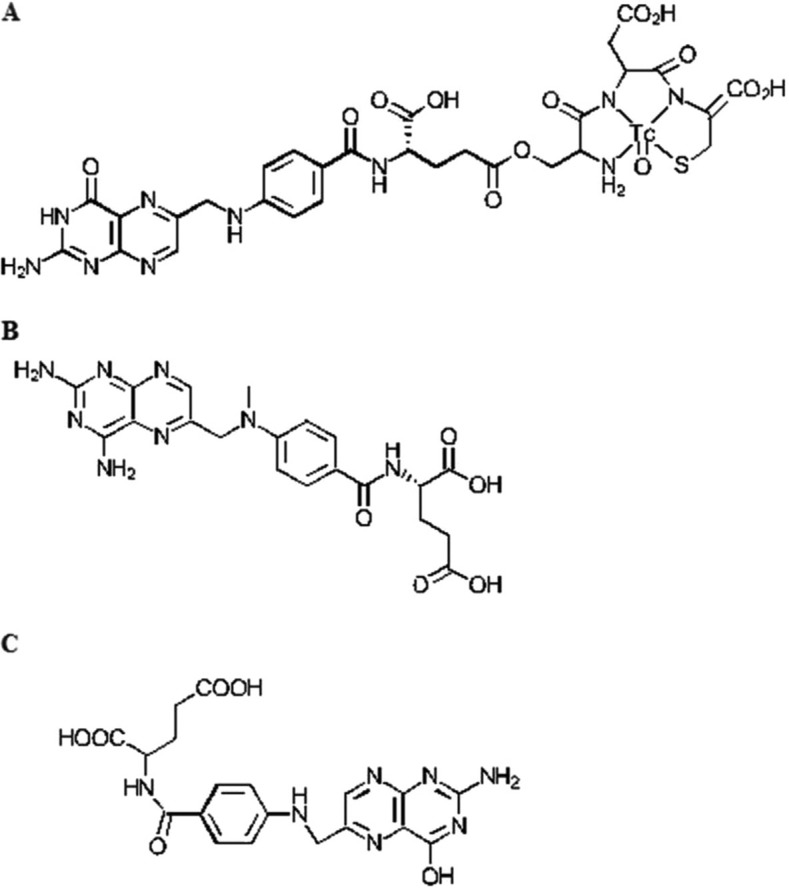
Structures of the molecules (A) ^99m^Tc-EC20, (B) methotrexate, and (C) folic acid.

**Fig. 4:**
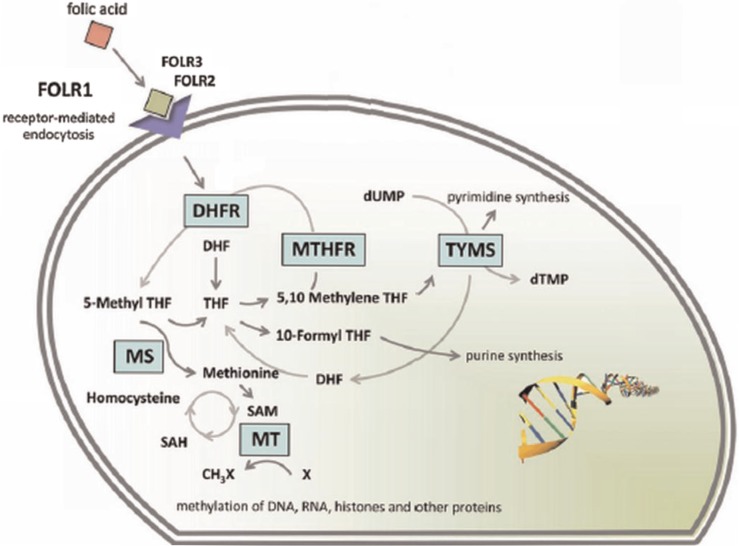
Overview of the folate cycle. The folate cycle is essential for rapidly growing cells. In cells, the endocytosis of folate is chiefly mediated through the alpha, beta, and gamma folate receptors (FOLR1-3) which have high affinities. (Reproduced with permission from Garcia-Bennett, Nees, Fadeel, 2011) (6).

**Fig. 5:**
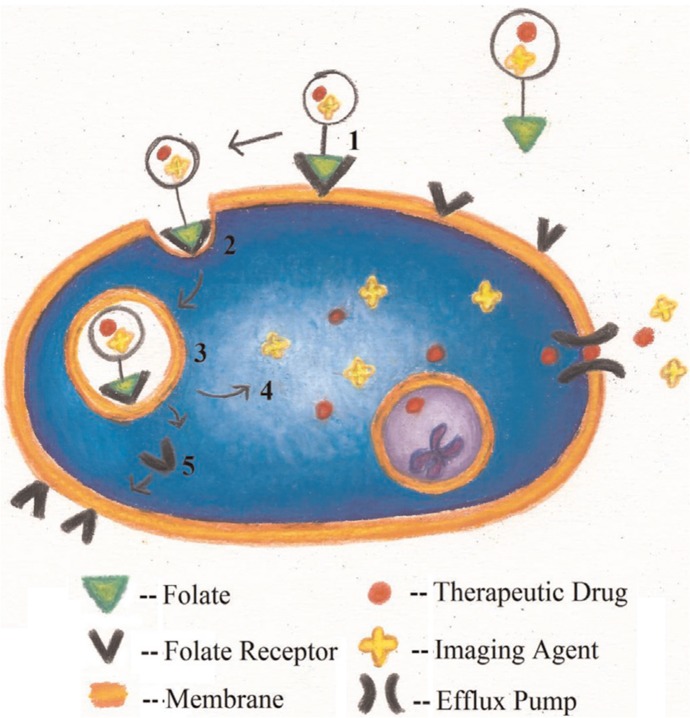
Receptor-mediated endocytosis of a drug conjugated to folate. (1) The drug–folate conjugate binds to folate receptors in caveolae. (2) Membrane invagination occurs and the drug–folate conjugate is engulfed by the cell and internalized by the endocytotic pathway. (3) Drug release is initiated by the low pH environment of the endosome. (4) Drug and imaging agents are released into the cell. (5) The folate receptor can be recycled and returned to the cell membrane. (Illustrated by Samantha L. Lindberg).

The folate receptor, a glycosylphosphatidylinositol anchored cell surface receptor, is overexpressed on the vast majority of cancer tissues, while its expression is limited in healthy tissues and organs (9). Folate receptors are highly expressed in epithelial, ovarian, cervical, breast, lung, kidney, colorectal, and brain tumors (10, 11). Sarcomas, lymphomas, and cancers of the pancreas, testicles, bladder, prostate, and liver often do not show elevated levels of folate receptors (10). When expressed in normal tissue, folate receptors are restricted to the lungs, kidneys, placenta, and choroid plexus (10). In these tissues, the receptors are limited to the apical surface of polarized epithelia (10). Folate, also known as pteroylglutamate, is a non-immunogenic water-soluble B vitamin that is critical to DNA synthesis, methylation, and repair (folate is used to synthesize thymine) (Figs. 3 and 4; 11–13). Folic acid is small (441 Da), stable over a broad range of temperatures and pH values, inexpensive, and non-immunogenic, and it retains its ability to bind to the folate receptor after conjugation with drugs or diagnostic markers (14). After folate attaches to the receptors located within caveolae, it is internalized through the endocytotic pathway. As the pH of the endosome approaches five, the folate dissociates from the receptor and the drug is released (Fig. 5; 13).

## Folate conjugates for cancer detection

The folic acid/folate receptor interaction can be targeted for imaging cancer cells by the attachment of imaging probe molecules to folate. Methods for detection of the probe/folate conjugates are non-invasive, making its use in locating and determining the severity of folate receptor–positive cancer very appealing. Current detection methods include optical imaging, magnetic resonance imaging (MRI), computer tomography, ultrasound imaging, single-photo emission computed tomography (SPECT) and positron emission tomography (PET) (15–17).

Presently MRI is one of the best molecular imaging methods available in a clinical setting because of its non-invasive nature, real-time monitoring, high spatial resolution, and multidimensional tomographic abilities (18–21). However, MRI has been plagued with low sensitivity issues (1). Because of the beneficial qualities of MRI, research has been aimed at improving the form of imaging. One step towards improving MRI has been through the use of iron oxide nanocrystals. Super-magnetic iron oxide nanoparticles (SPIONs) are widely used as contrast agents because they enhance negative contrast and allow for darker imaging of regions of interest (22–25; Fig. 2). The first SPIONs used were Ferumoxides (120–180 nm) for detections of lesions in the liver (6). The use of SPIONs for imaging liver tumors is a specialty use (26). It was found that Kupffer cells (hepatic macrophages) efficiently take up magnetic nanoparticles. Trade names for widely used SPIONs include Lumirem (ferumoxil, oral suspension), a bowel contrast agent, and Endorem, also known as Feridex, for spleen and liver imaging (27, 28). One of the greatest successes has come from Combidex, an ultra-small super-magnetic iron oxide nanoparticle (USPION). It is used in Europe for pinpointing lymph node metastases, but the USPIONs failed to pass United State Food and Drug Administration approval (29). Recent data suggest that folate-targeting magnetic core-shell nanocarriers, a class of SPIONs, may provide an effective cancer diagnostic nanomedical tool (30).

Carbon nanotubes are also being studied for use as MRI contrast agents (Fig. 2). In 2011, researchers tested folic acid conjugated functionalized multi-walled CNTS (MWCNTs) magnetic nanoparticle hybrids as contrast agents (17). The study results suggest that CNTs can be used as ideal targeted imaging agents that produce a strong MRI contrast. In another study, MWCNTs were targeted to cancer cells using the folate receptor using a novel imaging approach (31). Uniquely, the CNTs were identified using confocal Raman microscopy unlike the typically used confocal fluorescence microscopy, which relies on fluorescently labeled CNTs (31). Wang and colleagues (32) tested surface-enhanced Raman scattering nanoparticles in the detection and characterization of circulating tumor cells (CTC), which act as the pioneer cells of invasive cancer during the development of metastasis. In the study, a specific and sensitive method was successfully developed for detecting CTCs in peripheral blood specimens, providing insight into new ways of monitoring disease progression and therapy response along with a novel imaging procedure (32).

Another promising method of cancer detection is the use of quantum dots (QDs; Fig. 2) (1). QDs are inorganic–organic composite nanoparticles typically made of semiconductor materials in an inorganic transition metal core/shell system. Many QDs are composed of cadmium selenide (CdSe), cadmium telluride (CdTe), indium phosphide (InP), and indium arsenide (InAs) as core elements, and typically zinc sulfide (ZnS) as the shell (1). QDs confine electrons in three dimensions and so can be excited to emit light across the visible and infrared (IR) light spectrum (1). The main advantage of using QDs is that they can be excited with a single light source, while preserving the narrow emission of each individual particle (33). Up until recently, use of QDs has been problematic due to issues with stability and water dispersability. With development of new surface coatings, QDs now have increased stability and the ability to be functionalized with tumor targeting ligands (34–36).

In 2004, the first uses of QDs for targeted *in vivo* imaging were reported by Gao et al. (37). Since then, further studies have explored the use of QDs for cancer treatment and imaging. In 2009, Yang and colleagues tested folate receptor–targeted liposomes loaded with QDs for future cancer imaging applications (38). Based on the work by Yang and colleagues (38), researchers at the National University of Singapore used the folate receptor to target multifunctional (theranostic) liposomes containing both doxetaxel and QDs for cancer imaging and therapy (38, 39). It was found that compared to non-targeting liposomes, folate receptor–targeted liposomes had significantly higher cellular uptake and cytotoxicity (39).

Gold nanoparticles (AuNPs) are also being explored for their use in folate receptor–targeted cancer imaging. Being that they are easily functionalized, non-toxic, and non-immunogenic, AuNPs are seen as excellent candidates (40, 41). Gold nanorods strongly absorb and scatter light in the visible and NIR region, and have been tested as novel contrast agents (41–45).

Other methods also creating interest are SPECT and PET tracers. For micro-SPECT nuclear imaging, the most promising candidate label is ^99m^Tc-EC20, in which ^99m^Tc is complexed to folate through a short linker peptide (Cys-Asp-Dap-D-Glu-Pte) (Fig. 3) (14). The complex has been used to image several hundred patients. In an exploratory study performed on 155 patients with a variety of solid tumors, 68% exhibited uptake of ^99m^Tc-EC20 into their tumors (46). ^99m^Tc-EC20 was also found in the kidneys and bladders of most patients, consistent with expression of the folate receptor in those tissues (46). The uptake of radioactivity by the liver could be blocked by inhibitors of organic anion transport (46). The results of the study suggested that micro-SPECT with ^99m^Tc-EC20 is a safe, non-invasive procedure, without the need for biopsy, to identify recurrent or metastatic cancers that might respond well to folate receptor–targeted therapy (46).

**Fig. 6:**
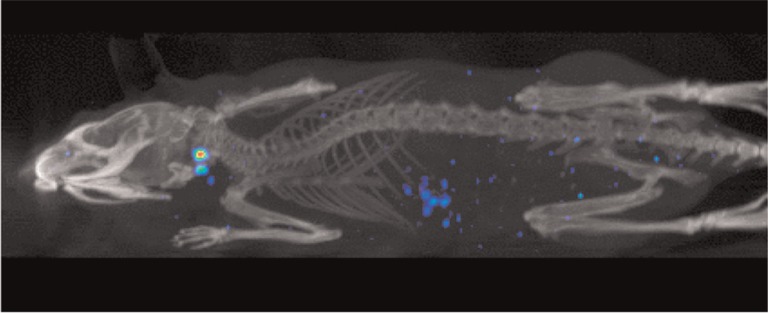
An example of an image resulting from a small animal that has undergone micro-SPECT imaging. The fluorescent blue areas indicate the locations of cancer cells. (Reproduced with permission from Bioscan) (67).

With PET nuclear imaging, development of folate based PET tracers would provide the most accurate method for non-invasive cancer diagnostics, especially with small metastases (14) (Fig. 6). However, recent studies had a significant drawback; a high intestinal, gallbladder, and kidney accumulation of radioactivity occurred with the PET folate derivatives tested (14). More recent data suggest that predosing with antifolate significantly reduces kidney uptake of folate PET tracers, while retaining radiotracer accumulation in cancerous tissues (14), but, as of now, unlike the ^99m^Tc-EC20 micro-SPECT tracer, which is now in clinical use, further developments in use of PET tracers are needed to take the applications from mice to human.

## Folate conjugates for cancer treatment

Until recently, the diagnosis and management of cancer have been seen as separate procedures. First generation nanoparticle therapies that have been approved by the FDA include the use of liposomes and micelles (47). Liposomes allow for the inclusion of drugs within their internal spaces, while micelles can encapsulate hydrophobic drugs (48). The largest group of nanoparticles includes organic, lipid, or polymeric nanomaterials (6). Organic-based nanoparticles have many terminal surface groups making functionalization and conjugation easy. For example, PEGylation, or the covalent attachment of polymeric ethylene glycol chains, is widely used, not to mention that PEG has been found to prevent recognition by the reticuloendothelial system, enabling longer systemic circulation (49, 50). Hydrophobicity is of major importance as it is a major hurdle in the delivery of poorly soluble drug compounds. But more recently, research has focused on the development of inorganic nanoparticles like gold (51).

By exploiting the unique properties of all nanoparticles, many of the already approved and currently studied nanomedical systems have proved to have multifunctional abilities. These multifunctional abilities combine the two previously separate notions of detection and treatment, rolling them into one process. Therefore, the drive for multifunctional clinical procedures has spurred the development of modifiable drug and imaging conjugates that are targeted to specific cells, thus allowing for simultaneous diagnosis and the controlled release of treatment drugs.

Active targeting of nanoparticles to specific cells has been proposed through the use of transferrin receptors, epidermal growth factor receptors (EGFR), folate receptors, human epidermal growth factor receptor two, integrins, somatostatin/growth hormone receptor (GH/GHR) and the glucose transporter (6, 44, 52–54). Of the candidates listed, the folate receptor has been extensively explored as a target for cancer treatments. Although, the EGFRs are expressed in head and neck cancers, glioblastomas, and lung adenocarcinomas, the folate receptor is rarely expressed there (55). The following technologies highlight successful and recent folic acid–mediated means of nanoparticle uptake for cancer treatment.

**Fig. 7:**
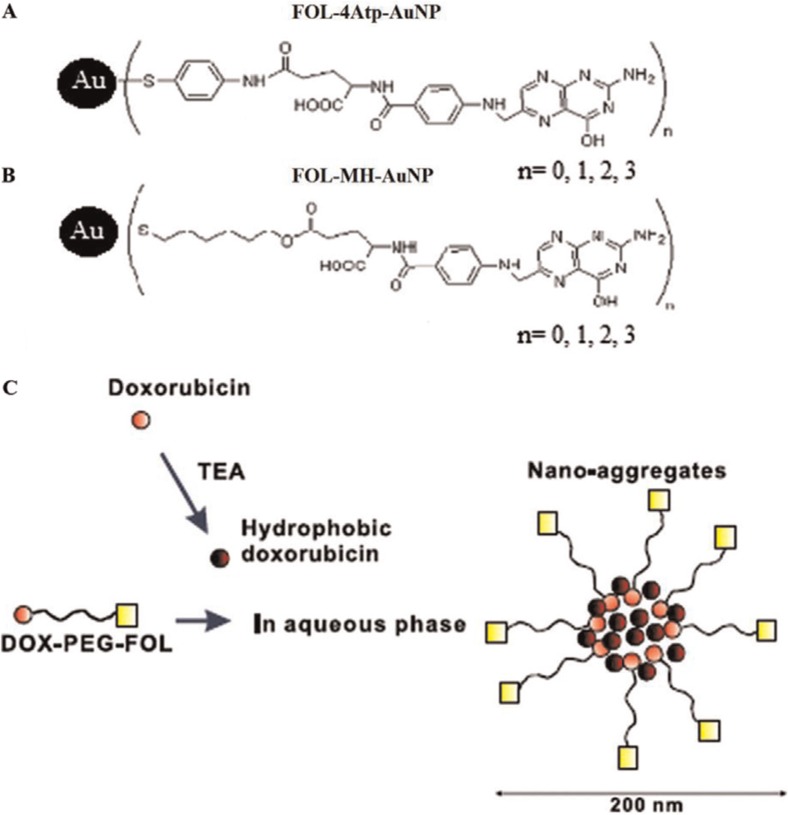
Structure of folate linked gold nanoparticles and doxorubicin-polyethylene glycol-folate nanoaggregates (A) folate-4-Aminothiophenol-gold nanoparticle (FOL-4Atp-AuNP) (B) folate-6-mercapto-1-hexanol-gold nanoparticle (FOL-MH-AuNP) (C) doxorubicin-polyethylene glycol-folate (DOX–PEG–FOL) nanoaggregates.

In a 2004 study performed at the Korean Advanced Institute of Science and Technology, the efficacy of nanoscale-sized folate receptor-targeted doxorubicin aggregates were tested for the treatment of cancer (9). The research team produced doxorubicin–polyethylene glycol–folate (DOX–PEG–FOL) conjugate micelles that were 200 nm in average diameter (Fig. 7). The polymeric micelles exhibited enhanced and selective targeting to folate receptor-positive cancer cells *in vitro*. More DOX–PEG–FOL nanoaggregates accumulated in folate receptor–positive human epidermal carcinoma KB cells than in folate receptor–negative A549 cells. When including unconjugated folate along with the nanoaggregates, the folate competitively inhibited binding of the DOX–PEG–FOL nanoaggregrates to the folate receptor–positive cells.

In *in vivo* animal experiments, the nanoaggregates caused significant tumor suppression. In human tumor xenograft nude mice, DOX–PEG–FOL nanoaggregates had a superior antitumor effect compared to other doxorubicin aggregates and free doxorubicin. In the mice treated with DOX–PEG–FOL nanoaggregrates, tumor volumes decreased by approximately 40% more than in mice treated with free doxorubicin. The enhanced antitumor effect of the nanoaggregrates was attributed to passive targeting through leaky vasculature in addition to active targeting of the nanoaggregates to folate receptors. Furthermore, the DOX–PEG–FOL nanoaggregates exhibited a sustained release effect because of prolonged circulation time in the bloodstream. Overall, the aggregates exhibited enhanced cellular uptake, increased targeting capacity, and increased cytotoxicity of folate receptor–positive cells.

While micelle nano-cancer treatments are still being tested in animal models, a study published in 2012 found 92% tumor growth inhibition (56). Uniquely, micelles were used to deliver meta-tetra(hydroxyphenyl)chlorin, a clinically used photosensitizer, to produce localized tissue damage using photodynamic therapy (56). The micelles reduced skin phototoxicity and lowered the effective dosage of photosensitizer by a third (56). Most frequently, studies have aimed at improving the delivery of paclitaxel (PTX) by loading the drug into micelles (57–59). The reasoning behind this is that PTX is a successful anticancer drug, but it lacks the ability to target cancer cells directly (57). Positive results of PTX loaded micelles include increased solubility, improved toxicity over free PTX, and lower systemic toxicity (57–59).

In a 2005 study at the University of Michigan Center for Biologic Nanotechnology and the Department of Radiation Oncology at the University of Michigan Health System, targeting of folate-linked methotrexate dendrimers was tested in immunodeficient athymic nude female mice and Fox Chase severe combined immunodeficient female mice (8). Mice were first injected with KB folate receptor–positive human cell lines. Tumors were allowed to grow for 2 weeks and reached a volume of 0.9 cm^3^. Then the mice were injected with the nanoconjugates twice a week via a lateral tail vein. Folic acid conjugates were delivered at an equimolar concentration with methotrexate, based on the number of methotrexate molecules present in each type of nanoparticle (Fig. 3). As a control experiment, the dendrimer was delivered at an equimolar concentration of the dendrimer in the conjugate. In the initial trial, the groups of mice received up to 15 injections. In the follow-up trial, mice received up to 28 injections, depending on survival. The dose of methotrexate injected each time equaled 0.33 mg/kg. Body weights were monitored as an indicator of adverse effects of the treatment. Tissues from the lungs, heart, liver, pancreas, spleen, kidney, and tumor were analyzed at the end of each trial. In addition, cells were isolated from tumors and stained with a targeted fluorescein-labeled conjugate to test for the presence of folic acid receptors.

The results from the study showed that conjugated methotrexate in dendrimers significantly lowered toxicity and resulted in a 10-fold higher efficacy compared to free methotrexate at an equal cumulative dose. Because of the ability to deliver a higher dose of methotrexate as the conjugate compared to the free drug, mice survived longer. However, the optimal dose of the targeted drug has not been definitively established because no toxic dose of the drug conjugate could be determined from either gross clinical evaluation or histopathology.

More recently, studies published using folate receptor–directed dendrimers have continued to investigate the delivery of methotrexate (60, 61). One study cited a 4,300-fold higher affinity for folate receptor–mediated methotrexate dendrimers than free drug alone (61). In 2012, researchers took a novel approach to dendrimer cancer treatment; dendrimers were used to deliver siRNA in order to improve its specificity and transfer activity (62). Results from the study indicated no inflammatory or interferon response, common non-specific effects of siRNA, suggesting future use as a potential cell-selective delivery method.

In a 2010 study at the University of Illinois at Chicago and the Department of Medical Physics at the Iran University of Medical Sciences in Tehran, researchers tested the efficacy of two folate conjugated gold nanoparticles for cancer treatment (13). The group actively targeted a gold nanosphere for use in the heat alabation of folate receptor–positive cancer cells. A combination of gold nanoparticles and an intense pulsed light, along with an incubation time, resulted in the significant death of cells with a high level of folate receptor expression and no significant cell death in cells with a low level of folate receptor expression.

The two conjugates that were used in the study include folate-4-aminothiophenol-gold nanoparticles (FOL-4Atp-AuNP) and folate-6-mercapto-1-hexanol-gold nanoparticles (FOL-MH-AuNP) (Fig. 7). Both conjugates have an absorption peak of ∼560 nm. Twenty pulses (3 ms) of intense pulsed light, with a wavelength of 560 nm, were used to heat the gold nanoparticles that were taken up by the cells that expressed a high level of folate receptors. During testing it was found that using up to 20 pulses of intense pulsed light had no harmful effects, and that nanoconjugate concentrations used in the study showed no toxicity. Treatments were evaluated at multiple time durations after heating.

Results from the study indicated that a longer treatment time is favorable over increased concentrations of the nanoconjugate. The highest level of cell death was observed after 4 h of incubation and 5 µg/mL of either nano-conjugate. The FOL-4ATP-AuNP was slightly more effective than the FOL-MH-AuNP at lower concentrations. The results show that a combination of gold nanoparticles and 20 pulses of intense ultraviolet (UV) light resulted in approximately 98% lethality of the cells expressing high level of folate receptors and only approximately 9% lethality of cells expressing a low level of folate receptors. The authors, however, did state that, for *in vivo* applications, IR light might be more effective than UV light as it penetrates deeper into tissues. Replacing the gold nanosphere moiety with nanoshells and nanorods, which absorb light more efficiently near IR wavelengths, should also be used for *in vivo* testing in the future. In addition, fiber optics might serve as an *in vivo* method for the deeper penetration of the light into the tissue.

Several other groups have also used mesoporous particles as targeted delivery agents (63, 64). In 2010, researchers found that mesoporous particles are well tolerated by mice, with a maximum dose of 100 mg/kg (63). In a 2012 study, Tabasi and colleagues tested the cytotoxicity of folate targeted mesoporous silicon doxorubicin drug conjugates (65). It was found that the mesoporous drug conjugates exhibited a substantially higher toxicity for tumor cells compared to free doxorubicin (65). Using folate as a targeting agent was clearly shown to enhance the toxicity of functionalized mesoporous silicon drug conjugates (65).

The ability of CNTs to be easily functionalized make them a promising candidate for cancer treatment. However, there are two major barriers to their use as cancer therapeutics. These include non-specificity and low potency (65). In 2010, Li and colleagues tested folate and iron difunctionalized MWCNTs for the delivery of doxorubicin into HeLa cells. The efficiencies of the drug conjugates were tested on HeLa cells in 96-well assays (66). The MWCNTs were shown to have sufficient load capacity and controlled release by near IT radiation (66). Results from this study demonstrated a six-fold increase in doxorubicin delivery compared to free doxorubicin alone (66).

## Discussion

Overall, nanotechnology is being explored for cancer prevention, detection, and treatment. Methods are being proposed and tested that could make diagnosis and treatment of cancer non-invasive and targeted directly to tumors. Nanoparticle conjugation with current drug technologies can result in reduced toxicity, and in some cases, a 10-fold higher efficacy than when the drug is administered without targeting. The ability to target only cancer cells may prove to eliminate adverse effects of treatment such as damage to the immune system and the loss of quick replicating cells.

The folate receptor appears to be a promising target for cancer imaging and treatment. Folate receptors are highly overexpressed on the surface of many tumor types. This expression can be exploited to target therapeutic compounds directly to cancerous tissues using many avenues. While these studies prove to be promising, the use of folate directed cancer treatments in human subjects still needs further development and testing. More work needs to be done to find the correct dosages and potential long-term effects of nanoparticle drug delivery for the treatment of cancer. Also, in countries that fortify foods with folic acid, or in people who take folic acid supplements, interactions between the effects of folate and antifolate need to be explored. Nevertheless, the successful use of folate conjugates indicates that receptor targeted nanoparticle treatments are a likely candidate for managing cancer.
